# Biopolymer-Based Films from Sodium Alginate and Citrus Pectin Reinforced with SiO_2_

**DOI:** 10.3390/ma15113881

**Published:** 2022-05-29

**Authors:** Luís Marangoni Júnior, Camila Rodrigues Fozzatti, Ewelina Jamróz, Roniérik Pioli Vieira, Rosa Maria Vercelino Alves

**Affiliations:** 1 Packaging Technology Center, Institute of Food Technology, Av. Brasil, 2880, Campinas 13070-178, Brazil; crfozzatti@gmail.com (C.R.F.); and rosa@ital.sp.gov.br (R.M.V.A.); 2 Department of Bioprocess and Materials Engineering, School of Chemical Engineering, University of Campinas, Campinas 13083-852, Brazil; ronierik@unicamp.br; 3 Department of Chemistry, Faculty of Food Technology, University of Agriculture, ul. Balicka 122, 30-149 Kraków, Poland; ewelina.jamroz@urk.edu.pl

**Keywords:** packaging, blend films, polysaccharides, reinforcing agents, nanosilica

## Abstract

Blend films based on sodium alginate (SA) and citrus pectin (P) reinforced with different concentrations of SiO_2_ (0–10% *w*/*w*) were developed in this study. From the morphological (SEM) and structural (FT-IR) evaluation, it was verified that the incorporation of the reinforcing agent did not drastically modify the microstructure of the films, nor did new chemical bonds form. However, the XRD results suggested a slight reduction in the crystallinities of the blends by the incorporation of SiO_2_. Among the formulations prepared, the addition of a 5% reinforcing agent was responsible for the simultaneous improvement of mechanical and barrier properties. Comparing the control sample (SA/P) with the SA/P/5.0%SiO_2_ film, the tensile strength increased from 27.7 ± 3.7 to 40.6 ± 4.5 MPa, and the water-vapor transmission rate decreased from 319.8 ± 38.7 to 288.9 ± 23.5 g m^−2^ day^−1^. Therefore, SiO_2,_ as a reinforcing agent in SA/P blends, represents a simple and effective strategy for improving the properties of biopolymer-based films in applications, such as packaging.

## 1. Introduction

Modern society is increasingly concerned with the use of fossil resources and environmental impact caused by the production and incorrect disposal of these materials. Within this context, packaging from fossil sources is important and widely used by society, as they are responsible for the containment, preservation, and distribution of products. Due to their low cost and favorable properties for the production of packaging, these plastics are consolidated in the globalized world [1,2]. Therefore, whenever possible, the use of natural, biodegradable, and renewable biopolymers should be considered for the production of packaging, as they can be alternatives in the reduction of environmental impact [1,3].

Biopolymers are materials obtained from substances derived from living organisms, and thus, an increasing number of biopolymers, such as polysaccharides and proteins, have been studied [3,4]. Polysaccharides have film-forming capacity due to their structural characteristics and they are being broadly applied in the production of biodegradable films [3]. Despite great promise regarding the application of biopolymers, these materials have high sensitivity to water vapor and poor mechanical properties, making their wide use in packaging difficult [5]. Consequently, ways to improve the properties of biopolymer-based films at a low cost have been created. Among them, the formulation of blends with different biopolymers and the incorporation of reinforcing agents are highlighted [5,6].

Among some of the promising candidates for the production of films based on biopolymers are sodium alginate and pectin. These polysaccharides were selected for this study due to their high availability in Brazil and low cost. Sodium alginate is obtained from brown algae (*Phaeophyceae*) that have high growth rates and are present on coasts with temperate climates, being greatly available to exploration [3,7,8]. Sodium alginate is an unbranched copolymer composed of mannuronic and glucuronic acid units and is widely used as a stabilizing thickener, gel-forming or film-forming agent in the pharmaceutical, medical, and food fields [9,10,11]. Pectin is present in the primary cell wall as well as the intercellular layer of plants, and is most commonly extracted from citrus peel, apple pomace, and beet pulp [12,13]. Pectin is a homopolymer composed mainly of galacturonic acid linked through α-1,4-glycosidic bonds and, similarly as alginate, is widely used in the food industry as a gelling, stabilizing, and thickening agent in products such as jellies, yogurt, fruity dairy drinks, and ice-cream [12,13,14]. Although sodium alginate and pectin are great candidates for the production of films in food packaging [15], these polysaccharides have poor mechanical properties and high sensitivity to water, making their widespread application in this area difficult [5,12].

The use of biopolymer blends is an alternative to improve properties and reduce the costs of films. Both alginate and pectin are of low cost, having nontoxic properties and demonstrating biodegradability, while showing some potential for application in biopolymer packaging [5,12]. To obtain a blend of 2 biopolymers, it is necessary to mix the compounds, resulting in a material with properties of both biopolymers. In this way, the production of blends is carried out to maximize the performance of a material, decreasing its sensitivity to water and improving its properties for a given application [16]. The combination of sodium alginate and pectin was investigated in other studies, giving rise to continuous and homogeneous films [17]. Furthermore, the inclusion of sodium alginate in pectin-based formulations improved the strength of the biopolymer network [18].

Nevertheless, in this context, the formulation of blends of different biopolymers, associated with the use of reinforcing agents, is an interesting strategy to improve film properties [5,19,20]. The use of reinforcing agents in films can result in improving their physical properties, such as tensile strength, thermal stability, and water-vapor barrier [10]. Some examples of commonly used inorganic reinforcing agents are TiO_2_, ZnO, and SiO_2_ [5,21]. Nanosilica (SiO_2_) has been widely studied in several polymeric systems and is found naturally in quartz sands, rocks, and clays [22]. However, it is necessary to know how these particles influence the organic matrix, and, therefore, research is necessary.

The incorporation of different SiO_2_ concentrations in sodium alginate or pectin films was investigated by evaluating the physical, chemical, and optical properties of the films [23,24,25]. Pectin films with SiO_2_ showed that nanoparticles can reduce water-vapor permeability by 30–60%, compared to films without nanoparticles [25]. Films with SiO_2_ concentrations between 0 and 8% were produced, the tensile strength and elongation at break being higher for the composition of 4.5% SiO_2,_ and the water-vapor barrier of the films reduced with increasing concentrations of SiO_2_ [23]. The application of low SiO_2_ concentrations (0 to 1.5%) in sodium alginate films resulted in better thermal stability of the films; nevertheless, this did not affect the morphological or barrier-related properties [24]. The mechanical and barrier properties of SiO_2_ embedded films depend on the concentration and dispersion of the reinforcing agents in the biopolymer matrix [23]. Thus, it is important to investigate which SiO_2_ concentration can improve the performance properties of each biopolymer matrix.

To the best of our knowledge, studies incorporating SiO_2_ as a reinforcing agent in a film based on sodium alginate and pectin have not yet been conducted. Therefore, to obtain materials with improved properties, the objective of this study was to produce biopolymer films composed of sodium alginate and pectin added to different concentrations of SiO_2_ and to characterize them in terms of their morphological, structural, and physicochemical characteristics.

## 2. Materials and Methods

### 2.1. Materials

The materials used in the preparation of the films were:

- Sodium alginate (SA) (Dinâmica Química Contemporânea Ltda., Indaiatuba/SP, Brazil) (purity of 99% viscosity (2% solution at 25 °C): 2.000 cps) as a film-forming compound;
- Pectin (P) powder from citrus (Dinâmica Química Contemporânea Ltda., Indaiatuba/SP, Brazil) (degree of methyl esterification: >75%) as a film-forming compound;
- Glycerol (Dinâmica Química Contemporânea Ltda., Indaiatuba/SP, Brazil) as a plasticizer; and
- Nanosilica (SiO_2_) (Degussa Brasil Ltda., Americana/SP, Brazil) as a strengthening component, with an average particle size of 12 nm.

### 2.2. Preparation of SA/P/SiO_2_ Films

The casting method was employed for the preparation of SA/P/SiO_2_ films. First, aqueous solutions of sodium alginate (2% *w*/*w*), pectin (2% *w*/*w*), glycerol plasticizer (30% *w*/*w* alginate + pectin) and SiO_2_ (0; 2.5; 5.0; 7.5 and 10.0% *w*/*w* alginate + pectin) were prepared. The compounds were dispersed using the Ultra-Turrax (model T18 basic, IKA^®^-Werke GmbH and Co. KG, Staufen, Germany) for 5 min at 7000 rpm. After preparation of the solution, it was heated up to 80 °C while stirring, and then maintained at this temperature for 15 min. Following, the solutions were homogenized again using the Ultra-Turrax for another 5 min at 7000 rpm. Subsequently, the final solutions were treated in an ultrasound bath for 15 min to remove bubbles. After cooling, 60 g of each solution was poured into glass Petri dishes (diameter equal to 15 cm) and then placed in an oven (Ethik Technology, Vargem Grande Paulista/SP, Brazil) at 40 °C for 24 h. Finally, the films were removed from the plates and placed in an air-conditioning chamber (Weiss Technik, Reiskirchen, Germany) at 25 °C and a relative humidity of 75% until the films were characterized.

### 2.3. Characterization of SA/P/SiO_2_ Films

#### 2.3.1. Film Morphology

The surface and cross-section of the SA/P/SiO_2_ films were analyzed using the method of scanning electron microscopy (SEM) (Leo 440i, LEO Electron Microscopy/Oxford, Cambridge, UK). The samples were previously fractured with liquid nitrogen, fixed on a metallic support using double-sided carbon tape and coated with gold in a sputter coater (SC7620, VG Microtech, Kent, UK). The visualization was performed with a magnitude of 1000×, voltage of 15 kV and current of 50 pA.

#### 2.3.2. Fourier Transform Infrared (FT-IR) Spectroscopy

FT-IR analysis was performed using the Thermo Scientific spectrometer (Nicolet Continuum, Madison, WI, USA). For all composite films, the attenuated total reflectance module (ATR) was used at 4000–675 cm^−1^, with a resolution of 4 cm^−1^ and 128 scans.

#### 2.3.3. Thermal Stability

Thermal stability testing was performed on 10 mg film samples heated from 25 to 600 °C at a nitrogen flow of 50 mL min^−1^ with a heating rate of 20 °C min^−1^. Analysis was performed using the Mettler Toledo Thermogravimetric Analyzer (TGA), model TGA/DSC1 (Schwerzenbach, Switzerland).

#### 2.3.4. X-ray Diffraction (XRD)

XRD measurements of composite films were performed with Cu Kα radiation (λ = 1.54056 Å) at a scan rate of 0.033333°/s (step = 0.04° and time per step = 1.2 s) with an accelerated voltage of 40 kV and an applied current of 40 mA, varying from 5 to 60°. The analysis was recorded on an X-ray analyzer (X’Pert-MPD, Philips, Almelo, The Netherlands).

#### 2.3.5. Thickness

Film thickness was measured using a micrometer (Mitutoyo, MDC-SX, Kawasaki, Japan). Five random locations around each film sample were used for thickness determination [26]. All tests were carried out with 5 repetitions.

#### 2.3.6. Mechanical Properties

The samples were cut to a width of 15 mm using high-precision equipment (RDS-100-C, ChemInstruments, Fairfield, OH, USA). Then, they were conditioned for 48 h at 23 ± 2 °C and 50 ± 5% RH. Tensile strength (TS), elongation at break (EB) and modulus of elasticity (ME) were determined using the curve generated from the software of a universal testing machine (Instron, 5966-E2, Norwood, MA, USA). The tests were performed with a 1 kN load cell at a speed of 12 mm min^−1^ and distances of 50 mm [27]. All tests were carried out with 5 repetitions.

#### 2.3.7. Water-Vapor Transmission Rate (WVTR)

The water-vapor transmission rate (WVTR) was determined by gravimetric analysis (3 replicates for each sample) using capsules (sealed by the films) with a 50 cm^2^ permeation area and analytical balance (Mettler Toledo, Columbus, OH, USA) with a resolution of 10^−4^ g. The tests were performed in an air-conditioned chamber (Weiss Technik, Reiskirchen, Germany) at 25 °C and 75% RH with anhydrous calcium chloride desiccant inside the capsule. The WVTR (g m^−2^ s^−1^) was determined from the slope of the curve “mass change vs. time” [28].

#### 2.3.8. Light Barrier

The light transmittance of the film samples was measured over a broad wavelength range (from UV to visible) between 200 and 800 nm using a UV-visible spectrophotometer (Cary 60, Agilent Technologies) with a scanning speed of 300 nm min^−1^ [29]. The tests were performed in triplicate.

#### 2.3.9. Statistical Analysis

The results were statistically evaluated using analysis of variance (ANOVA) and Tukey’s test to compare the means (*p* < 0.05).

## 3. Results and Discussion

### 3.1. Film Morphology

**Figure 1:**
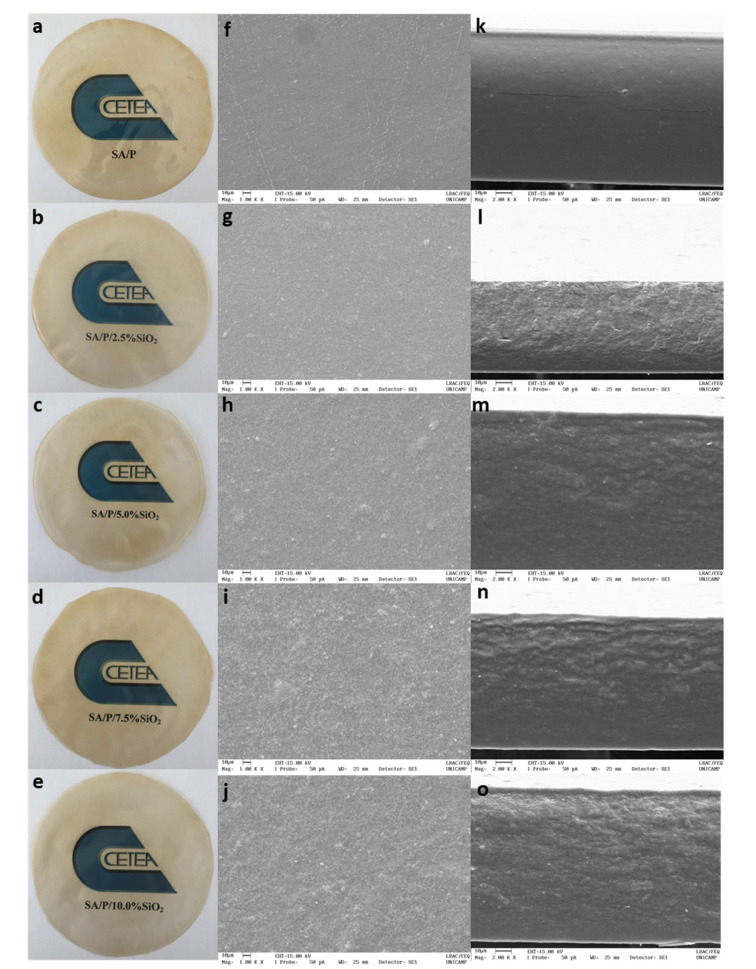
Photographs and SEM micrographs of the surface and cross-section of SA/P (**a**,**f**,**k**), SA/P/2.5%SiO_2_ (**b**,**g**,**l**), SA/P/5.0%SiO_2_ (**c**,**h**,**m**), SA/P/7.5%SiO_2_ (**d**,**i**,**n**), and SA/P/10.0%SiO_2_ (**e**,**j**,**o**) films.

The SA/P films were transparent and had a dark yellow color. The addition of SiO_2_ does not significantly affect the color of the film (Figure 1). SEM photos of the tested types of films are shown in Figure 1. SA/P films have an even, smooth surface without any cracks. However, cracks are visible in the cross-section, which may indicate slight compatibility between SA and P. Mallakpour and Mohammadi (2022), who also obtained films based on pectin and sodium alginate, reached similar conclusions [30]. The addition of SiO_2_ to the SA/P film changed the morphology of the matrix. An increase in the roughness of the film can be observed with a simultaneous increase in the concentration of SiO_2_. However, no accumulation of SiO_2_ in the film was noted. The type of biopolymer matrix used for SiO_2_ incorporation is of great significance. In the case of films based on sodium alginate and hydrolyzed collagen, the addition of SiO_2_ caused agglomeration of nanoparticles in the matrix, indicating poor dispersion and, consequently, the formation of a composite material instead of a nanocomposite [20]. Chitosan-SiO_2_ nanoparticles, formed at different times by ultrasonic treatment, were added to the thermoplastic starch and it was observed that the film surface became flatter while the size of the nanoparticles decreased. In addition, improvement in the mechanical properties was demonstrated [31]. The addition of SiO_2_ to the whey protein isolate/pullulan matrix made the structure compact and smooth [32]. Nonetheless, whether there is a dispersion of SiO_2_ in the biopolymer matrix or agglomeration of nanoparticles, in both cases, the mechanical properties of the presented films improved due to the presence of SiO_2_.

### 3.2. FT-IR Analysis

**Figure 2:**
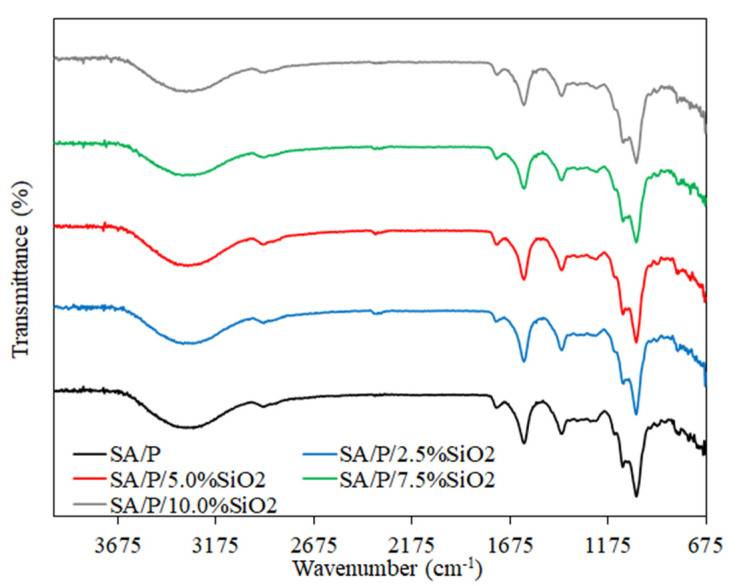
FT-IR spectra of SA/P and SA/P/SiO_2_ films.

The FTIR spectra for the SA/P films and their nanocomposites are presented in Figure 2. In general, the peaks related to pure biopolymers reported in previous studies were assigned [24,33]. The characteristic peaks for SA/P films were observed at 1097 and 1030 cm^−1^, which describe the O-H stretching vibration and glycosidic bonds linking 2 galacturonic sugar units, respectively [34]. Additionally, the peaks between 1400 and 1600 cm^−1^ are related to the COO^−^ asymmetric and symmetric stretching vibrations [20]. The broad band at approximately 3340 cm^−1^ corresponds to –OH stretching, which can be associated with the presence of hydroxyl groups from biopolymers and glycerol. The peak at 1740 cm^−1^ corresponds to the C=O stretching of aliphatic ester groups from P-based films [33]. The addition of SiO_2_ to the SA/P film did not cause any significant changes in the FTIR spectra, which may indicate that there was no chemical bonding between the components. The lack of changes in the spectrum can be attributed to the overlapping of the characteristic biopolymer and SiO_2_ bands [20,24].

### 3.3. XRD Analysis

**Figure 3:**
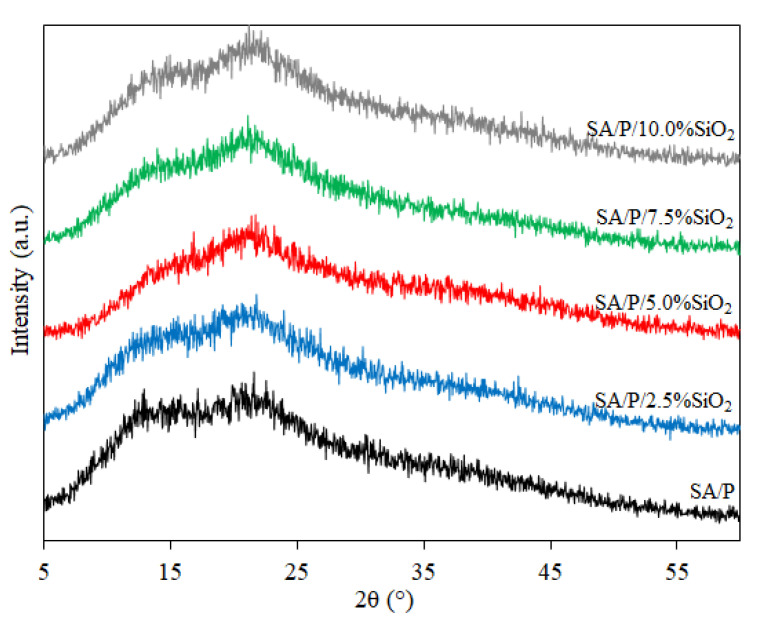
XRD patterns of SA/P and SA/P/SiO_2_ films.

In Figure 3, XRD patterns are provided for films from all samples evaluated in this study. Analyzing the profile of the SA/P control film, the presence of 2 broad peaks at 2θ ≈ 13° and 2θ ≈ 21° is verified, highlighting their mostly amorphous structure. This poor crystalline nature may also explain the presence of noise in all XRD results, which is commonly found in both SA [24] and P films [33]. As the SiO_2_ concentration increased, it could be seen that the broad peak at 2θ ≈ 13° reduced in intensity, indicating a slight reduction in the crystallinity of the blend film. Similar behavior was observed in the case of incorporating SiO_2_ in SA films [23] and adding montmorillonite clay to these films [35]. It is noteworthy that, even with the increase in SiO_2_ concentration, the typical broad and strong diffraction peak at 2θ ≈ 23° of its amorphous structure was not observed in any of the samples, confirming that there was good filler dispersion along the biopolymer matrix.

### 3.4. Thermal Stability of the Films

**Figure 4:**
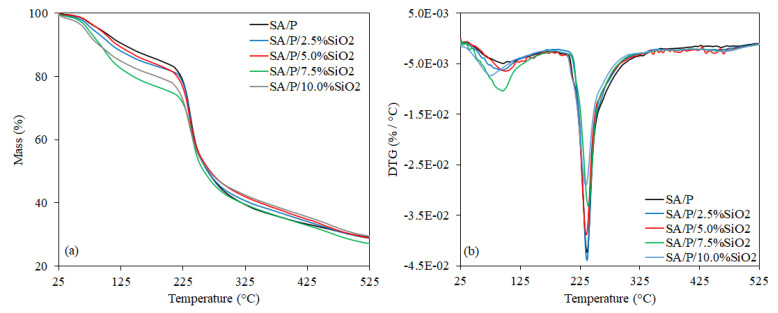
TGA (**a**) and DTG (**b**) of SA/P and SA/P/SiO_2_ films.

In Figure 4a,b, the TGA curves are presented, as well as their associated derivative (DTG) with 2 main thermal events. Additionally, in Table 1, the initial degradation temperature (*T_onset_*) is presented, as well as the temperature of maximum decomposition rate (*T_max_*), and mass loss (%) of these thermal events. The first thermal event, up to approximately 200 °C, is related to water evaporation. Water exists in 3 states within hydrophilic polymer matrices: (i) free water, which presents the same phase transition temperature as bulk water and becomes crystallized at 0 °C; (ii) freezable bound water, which crystallizes at a temperature lower than 0 °C due to the weak intermolecular forces between water molecules and polymeric chains, such as van der Waals interactions; and (iii) non-freezable bound water, which does not crystallize even when the sample is cooled to −100 °C. This last behavior is due to the strong hydrogen bonds between the polar moieties of hydrophilic polymers and water molecules [5,18,36,37]. In the thermal behavior profile of the present study, the mass loss at low temperatures can be ascribed to the elimination of free water, while above 100 °C, the release of freezable and non-freezable bound water may occur.

From Table 1, it is also possible to note that the incorporation of SiO_2_ apparently contributed to the higher moisture content of the films, which is associated with the higher percentages of mass loss in this first thermal event. This result apparently suggests a reduction in the thermal stability of the films. In the second thermal event, polysaccharide decomposition starts, involving random rupture of glycosidic bonds, vaporization, and elimination of volatile products [18]. In previous research, it has been demonstrated that the 2 polymers (SA and P) present similar depolymerization patterns, displaying very close thermal stability [18,33,38]. Furthermore, it was also shown that the incorporation of SiO_2_ increased the thermal stability of pure sodium alginate films [23,24,39] and sodium alginate/hydrolyzed collagen blend films [20].

However, when the second thermal event is evaluated (Figure 4a), it can be observed that the incorporation of SiO_2_ displayed different profiles. Apparently, there were no significant differences at the beginning of *T_onset_* when the SA/P, SA/P/2.5%SiO_2_, SA/P/7.5%SiO_2_ and SA/P/10.0%SiO_2_ samples were compared. However, there was noted improvement of *T_onset_* from 222.27 °C (SA/P film) to 241.47 °C for the SA/P/5.0%SiO_2_ film sample. The activation energies, determined using Broido’s method [40], were 85 kJ mol^−1^ (R^2^ = 0.993) and 74 kJ mol^−1^ (R^2^ = 0.991) for these samples, respectively, being within the range of 225–250 °C. The lower activation energy for SA/P/5.0%SiO_2_ indicates that less energy is needed to trigger its degradation process. Thus, despite having identified a larger *T_onset_*, it is not possible to state that the incorporation of SiO_2_ improved thermal stability during the second thermal event.

### 3.5. Thickness and Mechanical Properties

The control film (SA/P) had a thickness of 115.5 ± 13.0 µm (Table 2). The addition of SiO_2_ between 2.5% and 7.5% did not result in a significant difference in thickness with regard to the control film (*p* < 0.05). However, the addition of the highest concentration of SiO_2_ (10%) resulted in a significant increase in film thickness in relation to the other films. The increase in SA/P/10.0%SiO_2_ film thickness is due to the increase in dry matter from SiO_2_ in the polymer matrix, which is able to increase the free volume between macromolecular chains. Similar results were found for alginate/CuS films [3] and sodium alginate/hydrolyzed collagen/SiO_2_ films [20].

**Figure 5:**
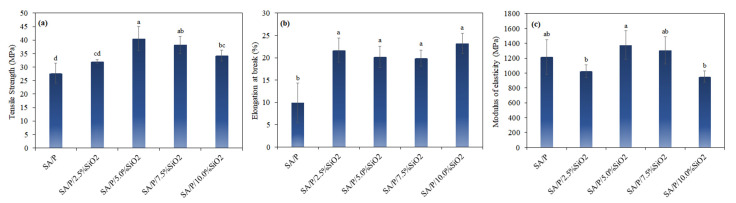
Mechanical properties of SA/P and SA/P/SiO_2_ films—(**a**) tensile strength, (**b**) elongation at break, and (**c**) modulus of elasticity. ^a, b, c, d^ The values followed by the same letter do not differ at the 95% confidence level (*p* > 0.05).

The control film (SA/P) showed a tensile strength (TS) of 27.7 ± 3.7 MPa (Figure 5a). The addition of SiO_2_ concentrations higher than 5% resulted in a significant increase in TS (*p* < 0.05). The highest TS was observed for the SA/P/5.0%SiO_2_ film, presenting a value of 40.6 ± 4.5 MPa. The increase in TS with the addition of SiO_2_ is related to the possible intermolecular interactions between SiO_2_ and the carboxylic groups of the polymer matrix, as previously observed for sodium alginate/hydrolyzed collagen/SiO_2_ films [20]. Despite the increase in TS, an increasing trend was not observed as a function of the rise in SiO_2_ content. This behavior may be related to the aggregation of SiO_2_ particles in the films with higher SiO_2_ loads, as observed in the SEM images (Figure 1).

The elongation at break (EB) of the control film (SA/P) was 9.9 ± 4.4%, while the films with the addition of SiO_2_ presented significantly higher values (*p* < 0.05), i.e., EB between 19.9 ± 1.9% and 23.2 ± 2.3% (Figure 5b). Regardless of the concentration, the films with SiO_2_ did not show significant differences between them concerning EB (*p* < 0.05). This behavior can be explained by the dispersion of SiO_2_ particles in the polymer matrix, causing mutual rearrangements of the chain units and resulting in better flexibility of the polymeric chains [41]. This result is in agreement with that obtained for sodium alginate/hydrolyzed collagen/SiO_2_ [20] and sodium alginate/agar/SiO_2_ films [41]. The modulus of elasticity (ME) of the control film (SA/P) showed an ME of 1215.3 ± 232.1 MPa, and the films with SiO_2_ addition did not present significantly different values (*p* < 0.05) (Figure 5c). Thus, the incorporation of SiO_2_ particles did not influence film stiffness.

In order to compare the obtained results regarding mechanical strength of the obtained films, in Table 3, the results are shown for the mechanical strength of films from other biopolymers. It may be noted that the obtained films have, in many cases, higher TS values than films obtained from other biopolymers, which also confirms the correct choice of material components.

### 3.6. WVTR

The results obtained from the water-vapor transmission rate (WVTR) of the SA/P/SiO_2_ films are presented in Table 2. The WVTR of the SA/P/2.5%SiO_2_ and SA/P/10.0%SiO_2_ films did not show any significant differences in relation to the control film (SA/P), with values varying between 319.8 ± 38.7 and 369.8 ± 30.0 g m^−2^ day^−1^. In other studies, low concentrations of SiO_2_ were also not sufficient to reduce the WVTR of K-carrageenan [44], sodium alginate [24] or sodium alginate/hydrolyzed collagen films [5]. On the other hand, films with excess SiO_2_ tend not to show a reduction in WVTR, as the high SiO_2_ charge can cause greater interaction with water molecules [41], as noted for the SA/P/10.0%SiO_2_ film.

The films with amounts of SiO_2_ fillers totaling 5.0 and 7.5% were effective in reducing the WVTR of the films, presenting values significantly lower than those for the control, with the lowest value equal to 276.5 ± 6.5 g m^−2^ day^−1^ obtained for the SA/P/7.5%SiO_2_ film. This reduction in the WVTR value may be associated with the formation of hydrogen bonds between the biopolymer matrix and the hydroxyls from the particles [32]. The strong interaction formed between SiO_2_ and the polymer matrix can be conducive to the formation of a denser matrix, preventing the passage of water molecules through the film [43]. In addition, the good dispersion of SiO_2_ particles contributes to the reduction of void spaces in the polymer matrix, consequently, reducing the WVTR of the SA/P films, as observed in the SEM images (Figure 1).

### 3.7. Light Barrier Properties

**Figure 6:**
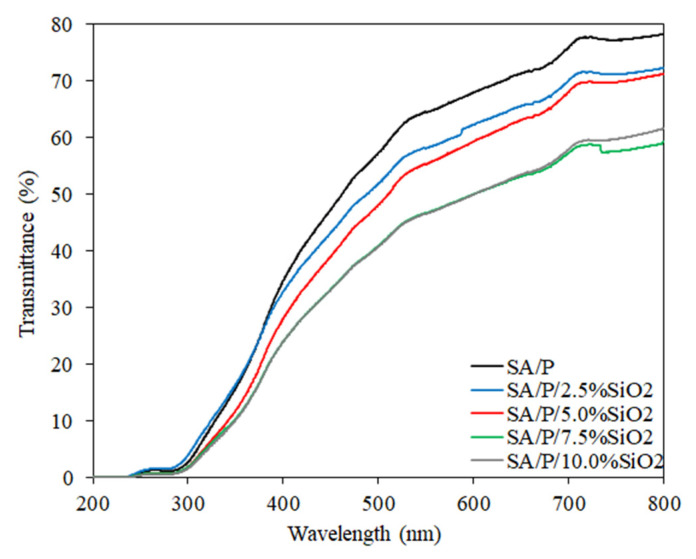
Light transmission profile (%) of SA/P and SA/P/SiO_2_ films.

The transmittance results as a function concerning the wavelength of the SA/P/SiO_2_ films are shown in Figure 6. The control film (SA/P) demonstrated higher transmittance within the visible light wavelength range (400–800 nm) than films incorporated with SiO_2_. The reduction in visible light transmission as the concentration of SiO_2_ increased may be related to light scattering caused by the incorporation of particles at the nanoscale [23]. Considering the ultraviolet (UV) region, particularly between 200 and 300 nm, the transmittance was very close to 0 for all samples. The biopolymer molecules that make up the films themselves comprise chromophore groups with high UV-light absorption capacity. For example, P-based films presented a UV light-barrier effect up to 300 nm related to n→π∗ transitions in carbonyl groups of carboxylate or ester moieties [33], which may also be present in the SA behavior. Hence, the addition of SiO_2_ had no apparent, significant influence on this result. Furthermore, for all films, regardless of SiO_2_ concentration, the UV-light barrier profiles were similar even at wavelengths between 300 and 400 nm. The reduction of transmittance occurred, but at a very little pronounced intensity.

## 4. Conclusions

The aim of this study was to investigate the effects of incorporating different concentrations (0–10%) of SiO_2_ on the physicochemical properties of blend films based on natural polymers: sodium alginate (SA) and citrus pectin (P). Overall, it was concluded that the most suitable formulation contained 5% SiO_2_, as it enabled simultaneous improvement of mechanical, barrier-related and thermal properties. Comparing the control sample (SA/P) with the SA/P/5.0%SiO_2_ one, tensile strength increased from 27.7 ± 3.7 to 40.6 ± 4.5 MPa, elongation at break increased from 9.9 ± 4.4 to 19.9 ± 1.9%, while modulus of elasticity did not present significant alteration. Thus, it was possible to improve the strength of the film and its plasticity without drastically modifying its rigidity. In parallel, the WVTR decreased from 319.8 ± 38.7 to 288.9 ± 23.5 g m^−2^ day^−1^ for the same formulation with 5% SiO_2_, indicating an increased moisture barrier. It is also important to highlight that there was an increase in the light barrier of the films as a function of SiO_2_ concentration, suggesting applicability in the protection of foods subject to deterioration caused by this variable. Therefore, SiO_2_ as a reinforcing agent in SA/P blends, represents a simple, inexpensive and effective strategy for improving the properties of biopolymer-based films.

## Figures and Tables

**Table 1:** Onset temperature (*T_onset_*), temperature of maximum degradation rate (*T_max_*) and mass loss of SA/P and SA/P/SiO_2_ films.

	First Thermal Event			Second Thermal Event		
Film	*T_onset_* (°C)	*T_max_* (°C)	Mass Loss (%)	*T_onset_* (°C)	*T_max_* (°C)	Mass Loss (%)
SA/P	54.24	105.74	14.22	222.27	243.52	49.06
SA/P/2.5%SiO_2_	51.47	104.44	16.69	222.33	241.93	44.92
SA/P/5.0%SiO_2_	64.34	107.67	15.89	241.47	240.27	42.26
SA/P/7.5%SiO_2_	55.16	97.04	22.90	226.41	242.58	40.27
SA/P/10.0%SiO_2_	49.51	92.00	19.20	219.97	242.63	40.69

**Table 2:** Thickness and water-vapor transmission rate (WVTR) of SA/P and SA/P/SiO_2_ films.

Film Sample	Thickness (µm)	WVTR (g m^−2^ day^−1^)
SA/P	115.5 ± 13.0 ^b^	319.8 ± 38.7 ^a^
SA/P/2.5%SiO_2_	115.6 ± 7.6 ^b^	338.7 ± 19.0 ^a^
SA/P/5.0%SiO_2_	116.0 ± 13.0 ^b^	288.9 ± 23.5 ^b^
SA/P/7.5%SiO_2_	119.5 ± 15.7 ^b^	276.5 ± 6.5 ^b^
SA/P/10.0%SiO_2_	126.6 ± 13.0 ^a^	369.8 ± 30.0 ^a^

The results are expressed as an average ± standard deviation. ^a, b,^ The values in a given column followed by the same letter do not differ at the 95% confidence level (*p* > 0.05).

**Table 3:** Influence of SiO_2_ additive on mechanical properties (tensile strength- TS and elongation at break-EAB) of selected biopolymer films.

Type of Films	TS [MPa]	EAB [%]	Reference
Whey protein isolate/pullulan	2.3–3.1	122.0–162.3	[32]
Chitosan	30.3–38.4	17.7–22.6	[42]
Sodium alginate/hydrolyzed collagen	20.2–25.4	30.2–35.8	[20]
Sodium alginate	20.4–30.6	20.0–29.3	[24]
Carrageenan	6.1–10.3	9.0–19.5	[43]

## Data Availability

Not applicable.
